# Supplementary material for: Antigen presentation by B cells enables epitope spreading across an MHC barrier
Source: Nat Commun. 2023 Oct 31;14:6941. doi: 10.1038/s41467-023-42541-7 (PMC10618542; doi:10.1038/s41467-023-42541-7)
Supplement: Supplementary file 1 — Supplementary Information [file 41467_2023_42541_MOESM1_ESM.pdf]

Supplementary Information

Antigen presentation by B cells enables epitope spreading across an MHC barrier

Fahlquist-Hagert et al.

Table of Contents:

Page 1: Index
Page 2: Supplementary Figure 1
Page 4: Supplementary Figure 2
Page 5: Supplementary Figure 3
Page 7: Supplementary Figure 4
Page 9: Supplementary Figure 5
Page 11: Supplementary Figure 6
Page 13: Supplementary Figure 7
Page 14: Supplementary Figure 8
Page 16: Supplementary Figure 9
Page 18: Supplementary Figure 10
Page 20: Supplementary Figure 11
Page 21: Supplementary Figure 12
Page 22: Supplementary Table 1
Page 24: Supplementary Table 2

Supplementary Figures

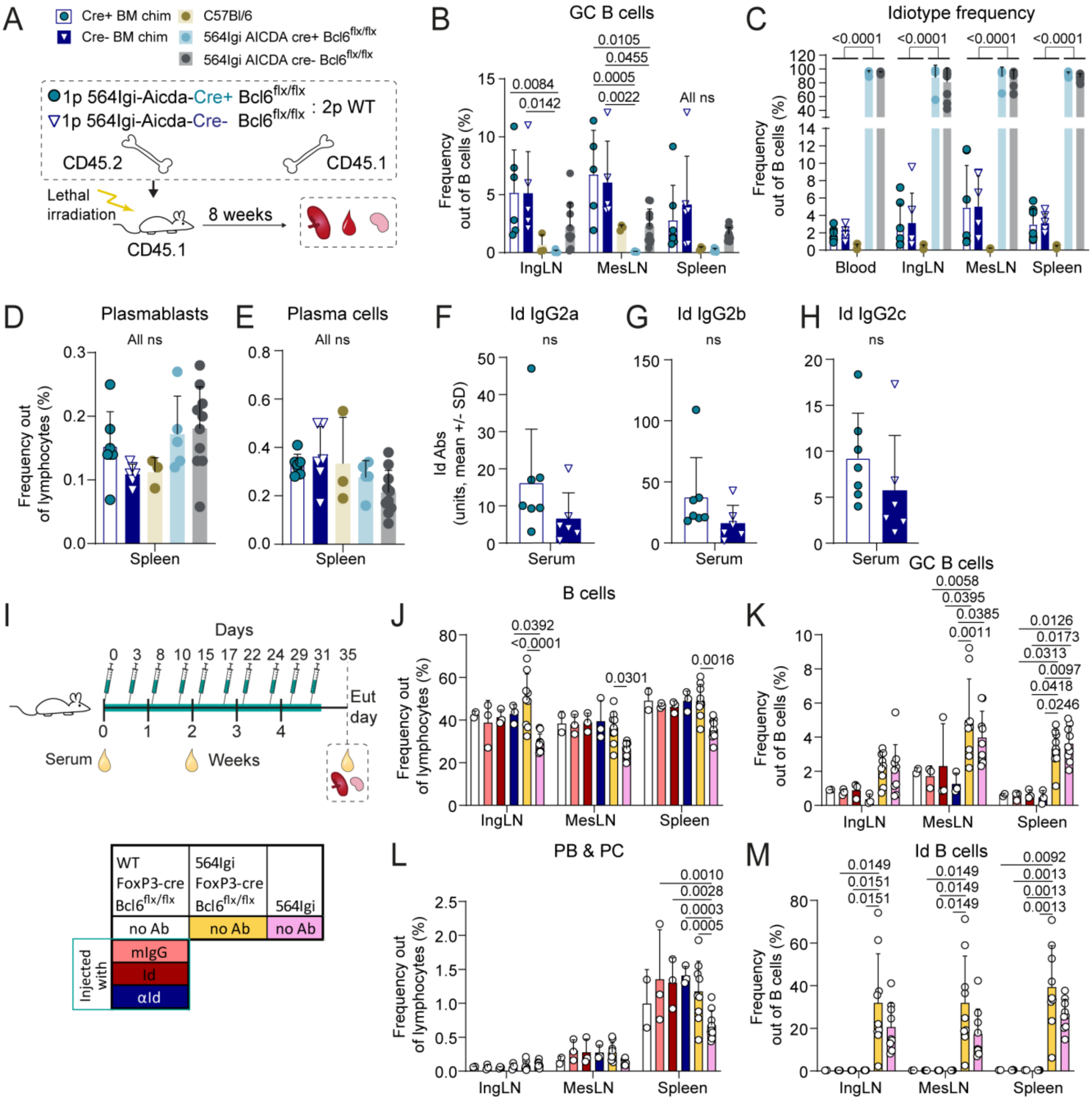

Supplementary Figure 1. Supporting data for main Figures 2 and 3. (A) Schematic overview of experimental setup, including additional biological controls as indicated in the expanded legend, i.e., wt C57Bl/6J, and homozygous 564Igi Bcl6$^{flx/flx}$ either positive (AICDA cre+) or negative (AICDA cre-) for Cre under the control of the *Aicda* promotor. (B) Frequency of GC B cells in blood, spleen, inguinal and mesenteric lymph nodes (IngLN and MesLN, respectively) of Aicda-Cre+ (circles, clear bars, n=7) and Aicda-Cre- (triangles, blue bars, n=6) Bcl6$^{flx/flx}$ 564Igi mixed chimeras, C57Bl/6J mice (brown circles, light brown bars, n=3), Aicda-Cre+ Bcl6$^{flx/flx}$ homozygous 564Igi mice (light blue circles and bars, n=5), or Aicda-Cre- Bcl6$^{flx/flx}$ homozygous 564Igi mice (dark grey circles and light gray bars, n=10). Bars indicate mean+/-SD and p-values are given for two-way ANOVA with Tukey's post-test. (C) as B, but for idiotype frequencies, and including blood as well. (D) as B, but for plasmablasts and only in

spleen, with statistical significance based on one-way ANOVA with Tukey's post-test. (E) as D, but for plasma cells. (F) IgG2a idiotype antibody levels in serum of Aicda-Cre+ (circles, clear bars, n=7) and Aicda-Cre- (triangles, blue bars, n=6) Bcl6$^{flx/flx}$ 564Igi mixed chimeras. Bars indicate mean+/-SD and p-values are given are for unpaired, two-tailed t-test with Welch's correction. (G) as F, but for IgG2b. (H) as F, but for IgG2c. (I) Schematic overview of experimental setup, including additional biological controls as indicated in the expanded legend, i.e., heterozygous 564Igi FoxP3-cre Bcl6$^{flx/flx}$ mice or regular heterozygous 564Igi mice. (J) Frequency of B cells in IngLN, MesLN and spleen of wt FoxP3-cre Bcl6$^{flx/flx}$ non-injected (clear bars, n=2), injected with murine IgG (light red bars, n=3), Id antibody (dark red bars, n=3), or anti-Id antibody (blue bars, n=3), non-injected heterozygous 564Igi FoxP3-cre Bcl6flx/flx (yellow bars, n=9), or non-injected heterozygous 564Igi mice (pink bars, n=9). Bars indicate mean+/-SD and p-values are given for two-way ANOVA with Tukey's post-test. (K) as J, but for GC B cells. (L) as J, but for PB+PC. (M) as J, but for Idiotype B cells.

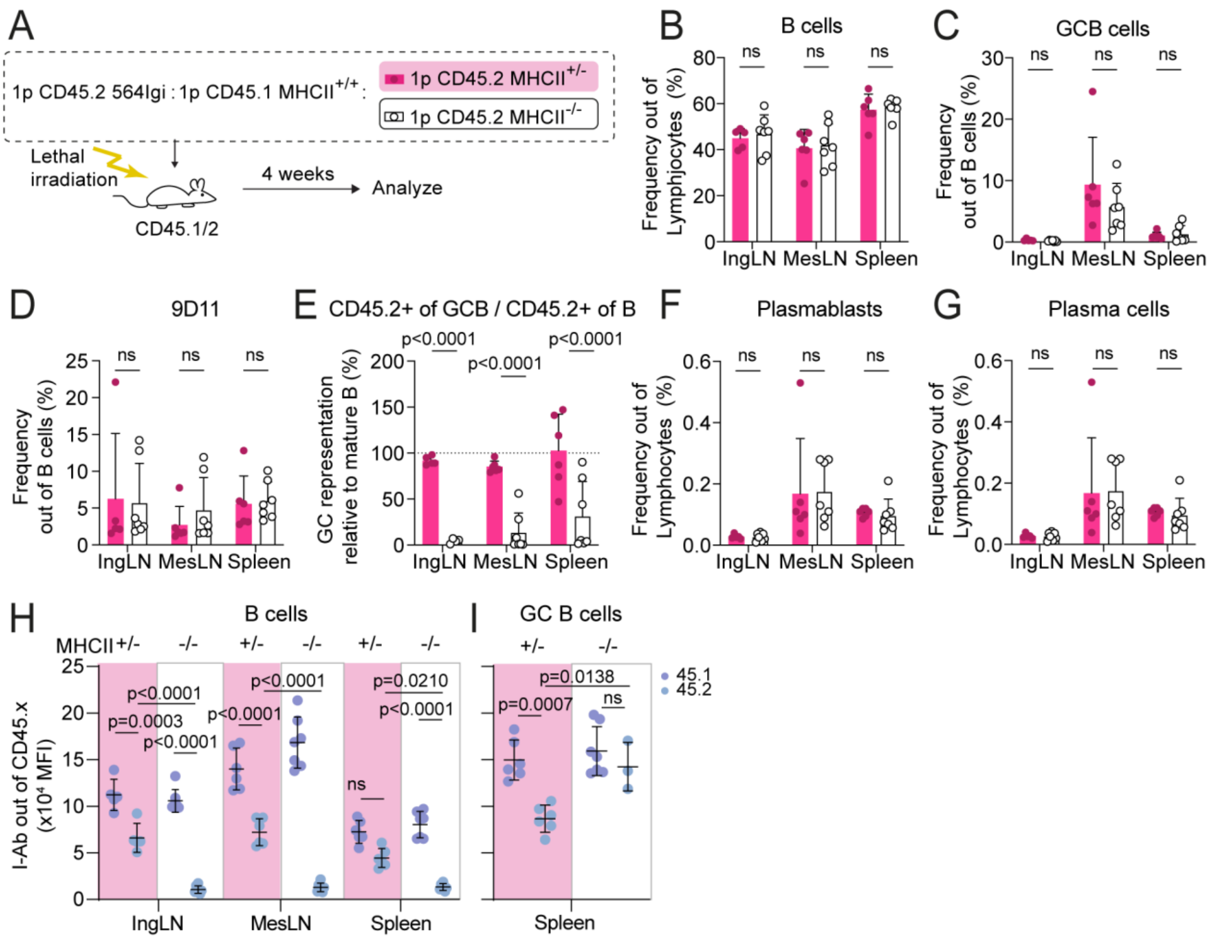

Supplementary Figure 2. Inclusion of the WT compartment absolutely requires MHCII. (A) Experimental setup. 1p = 1 part. Frequency of B (B) and GC B (C) cells in IngLN, MesLN and spleen of bone marrow chimeras. (D) Representation of CD45.2/2 cells in GCs relative to their representation within the total B cell compartment. Frequency of Id+ B cells (E), plasmablasts (F), and plasma cells (G). MFI of I-Ab for CD45.1 vs. CD45.2 B cells (H) or GC B cells (I) across lymphoid tissues of MHC$^{+/-}$ and MHC$^{-/-}$ chimeras. Bars and error bars signify mean±SD, for n=6 (MHC$^{+/-}$) and 7 (MHC$^{-/-}$) chimeras, in all graphs. Two-way ANOVA with Šidák's post-test used for comparisons in B-I. ns = p > 0.05.

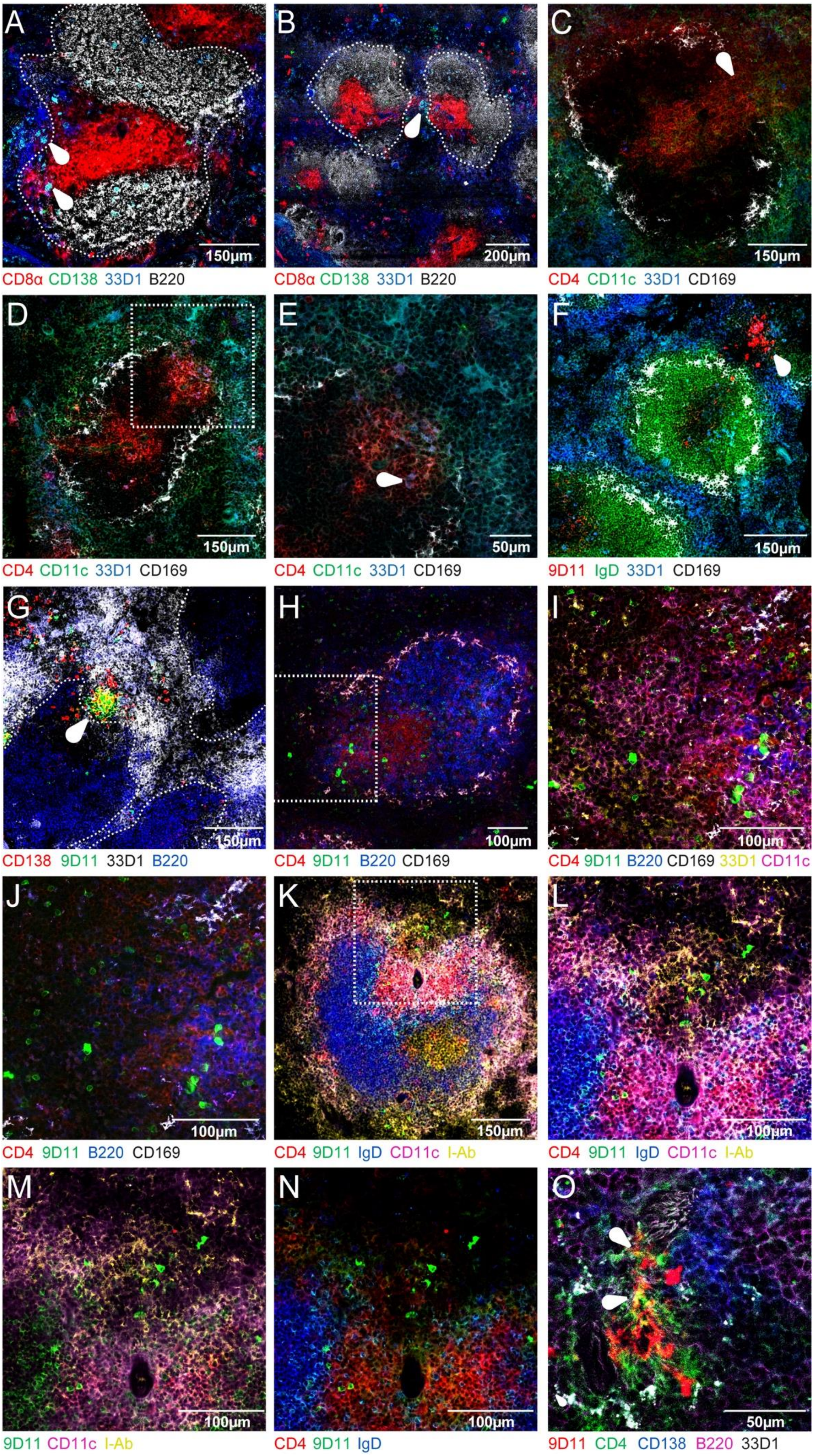
A
150µm
CD8α CD138 33D1 B220
B
200µm
CD8α CD138 33D1 B220
C
150µm
CD4 CD11c 33D1 CD169
D
150µm
CD4 CD11c 33D1 CD169
E
50µm
CD4 CD11c 33D1 CD169
F
150µm
9D11 IgD 33D1 CD169
G
150µm
CD138 9D11 33D1 B220
H
100µm
CD4 9D11 B220 CD169
I
100µm
CD4 9D11 B220 CD169 33D1 CD11c
J
100µm
CD4 9D11 B220 CD169
K
150µm
CD4 9D11 IgD CD11c I-Ab
L
100µm
CD4 9D11 IgD CD11c I-Ab
M
100µm
9D11 CD11c I-Ab
N
100µm
CD4 9D11 IgD
O
50µm
9D11 CD4 CD138 B220 33D1

Supplementary Figure 3. The initiating clone localizes to extrafollicular splenic bridging channels at time of GC establishment. (A) Representative spleen section stained for CD8α (red), CD138 (green), 33D1 (DCIR2, blue), and B220 (white). Arrows indicate juxtaposition of CD138 and 33D1 cells at the border of the marginal zone (MZ, dashed white line based on CD169/MOMA-1 staining). (B) As N, but zoomed out view of another section. (C) Staining for CD4 (red), CD11c (green), 33D1 (DCIR2, blue), and CD169 (MOMA-1, white). Arrow indicates area where the MZ is disrupted, and the T cell zone directly borders the red pulp. (D) As C. Box indicates area of zoom-in presented in panel E. (E) Arrow indicates juxtaposition of CD4 and 33D1 (DCIR2). (F) Staining for 9D11 (Idiotype, red), IgD (green), 33D1 (DCIR2, blue), and CD169 (MOMA-1, white). Arrow indicates prominent Id+ cells in the red pulp. (G) Staining for CD138 (Syndecan-1, red), 9D11 (green), 33D1 (DCIR2, white), and B220 (blue). Arrow indicates prominent CD138 and idiotype double-positive cells in the red pulp bordering the MZ (dashed white line based on CD169/MOMA-1 staining). (H) Zoom-out showing CD4, B220, CD169 and 9D11 after staining for CD4 (red), 9D11 (Idiotype, green), B220 (blue), CD169 (MOMA-1, white), 33D1 (DCIR2, yellow), and CD11c (magenta). Notice the break in the MZ (white box), and the position of 9D11 bright cells in this area. (I) As H, but zoom-in showing all channels. (J) as I, but showing only CD4, 9D11, B220 and CD169 for clarity. (K) Staining for CD4 (red), 9D11 (Idiotype, green), IgD (blue), I-Ab (MHCII, yellow), and CD11c (magenta). Zoom-out showing disruption of MZ by I-Ab and 9D11 cells proximal to the periarteriolar lymphoid sheath (white box). (L) Zoom-in of area indicated in K. (M) Zoom-in as in L, but showing only 9D11, I-Ab and CD11c for clarity. (N) as M, but zoom-in showing CD4, 9D11, IgD. (O) High-resolution image showing CD4 (red), 9D11 (Idiotype, green), CD138 (Syndecan-1, blue), B220 (magenta), and 33D1 (DCIR2, white). Arrows indicate close approximation, or even partial overlap of CD4 and 9D11 staining. Color intensities adjusted uniformly for visual clarity in micrographs.

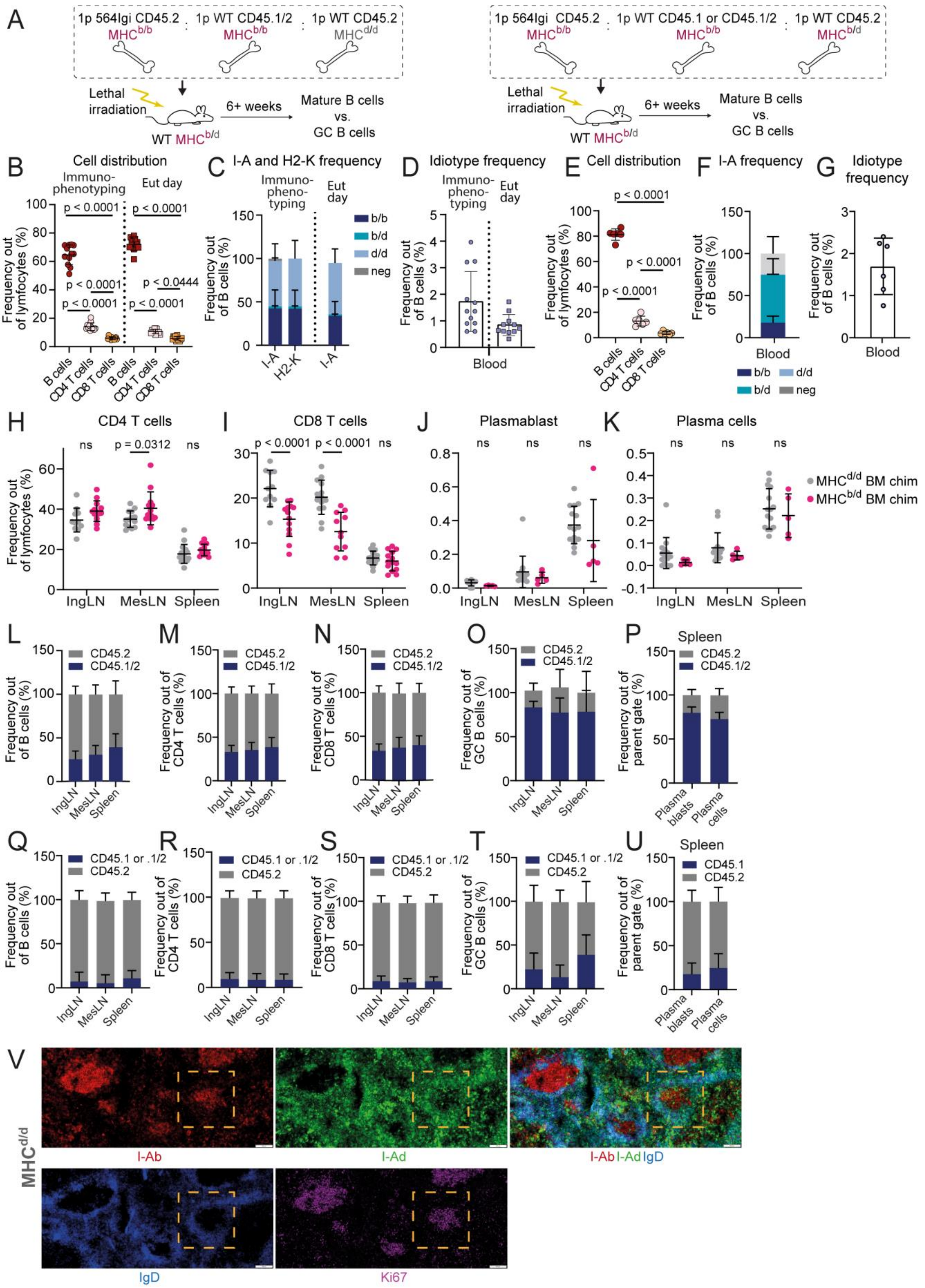
A
1p 564Igi CD45.2 : 1p WT CD45.1/2 : 1p WT CD45.2
MHCb/b MHCb/b MHCd/d
Lethal irradiation
6+ weeks
WT MHCb/d
Mature B cells vs. GC B cells
1p 564Igi CD45.2 : 1p WT CD45.1 or CD45.1/2 : 1p WT CD45.2
MHCb/b MHCb/b MHCb/d
B Cell distribution
C I-A and H2-K frequency
D Idiotype frequency
E Cell distribution
F I-A frequency
G Idiotype frequency
Immuno-phenotyping
Eut day
Frequency out of lymfocytes (%)
Frequency out of B cells (%)
p < 0.0001
p < 0.0444
B cells
CD4 T cells
CD8 T cells
b/b
b/d
d/d
neg
Blood
H CD4 T cells
I CD8 T cells
J Plasmablast
K Plasma cells
ns
p = 0.0312
MHCd/d BM chim
MHCb/d BM chim
IngLN
MesLN
Spleen
L
M
N
O
P Spleen
CD45.2
CD45.1/2
Frequency out of CD4 T cells (%)
Frequency out of CD8 T cells (%)
Frequency out of GC B cells (%)
Frequency out of parent gate (%)
Plasma blasts
Plasma cells
Q
R
S
T
U Spleen
CD45.1 or .1/2
CD45.1
V
MHCd/d
I-Ab
I-Ad
I-Ab I-Ad IgD
IgD
Ki67

Supplementary Figure 4. Supporting data for chimeras in main Figure 4. (A) Schematic overview showing the two BM chimera setups to evaluate the importance of MHC haplotype for B cell participation in germinal centers (GCs). 1p = 1 part. Distribution of B, CD4 T and CD8 T cells (B), I-A and H2-K haplotype frequencies (C), and idiotype frequencies (D) in blood of MHC$^{d/d}$ chimeras at 7-8 weeks (immunophenotyping) or 10-16 weeks (euthanasia day, eut day) post reconstitution. Distribution of B, CD4 T and CD8 T cells (E), I-A and H2-K haplotype frequencies (F), and idiotype frequencies (G) in blood of MHC$^{b/d}$ chimeras at 6-7 weeks (immunophenotyping) post reconstitution. Frequencies of CD4 (H), CD8 (I) T cells, plasmablasts (J) and plasma cells (K) out of lymphocytes in inguinal and mesenteric lymph nodes (IngLN and MesLN, respectively), and spleen. Ratio of CD45.1/2 and CD45.2 positive cells within the B cell gate (L), of CD4 (M), CD8 (N) T cells and GC B cells (O) of IngLN, MesLN and spleen for MHC$^{d/d}$ chimeras. (P) As I, but for the plasmablast/plasma cell gates of spleen only. (Q) As I, but for MHC$^{b/d}$ chimeras. (R) As J, but for MHC$^{b/d}$ chimeras. (S) As K, but for MHC$^{b/d}$ chimeras. (T) As L, but for MHC$^{b/d}$ chimeras. (U) As M, but for MHC$^{b/d}$ chimeras. (V) Representative crop-out of tile scan of spleen from an MHC$^{d/d}$ chimera, where a low number of I-A$^{d/d}$ cells have infiltrated an otherwise I-A$^{b/b}$ dominated GC (yellow dashed box). Left, single channel views of I-A^{b} (red), I-A^{d} (green), IgD (blue) and Ki67 (purple); right, overlay of all I-A^{b}, I-A^{d} and IgD. Color intensities were adjusted uniformly for visual clarity. Scale bars represent 100 µm. The d/d BM chimeras were repeated 2 times, the b/d BM chimeras were repeated 3 times. Error bars represent mean±SD, n=14-15 mice per group. Statistical significance given for two-way ANOVA with Šidák's post-test throughout, except for D, which is based on unpaired, two-tailed t-test with Welch's correction. ns: $p>0.05$. The markers for plasmablasts and plasma cells were only included in one experiment.

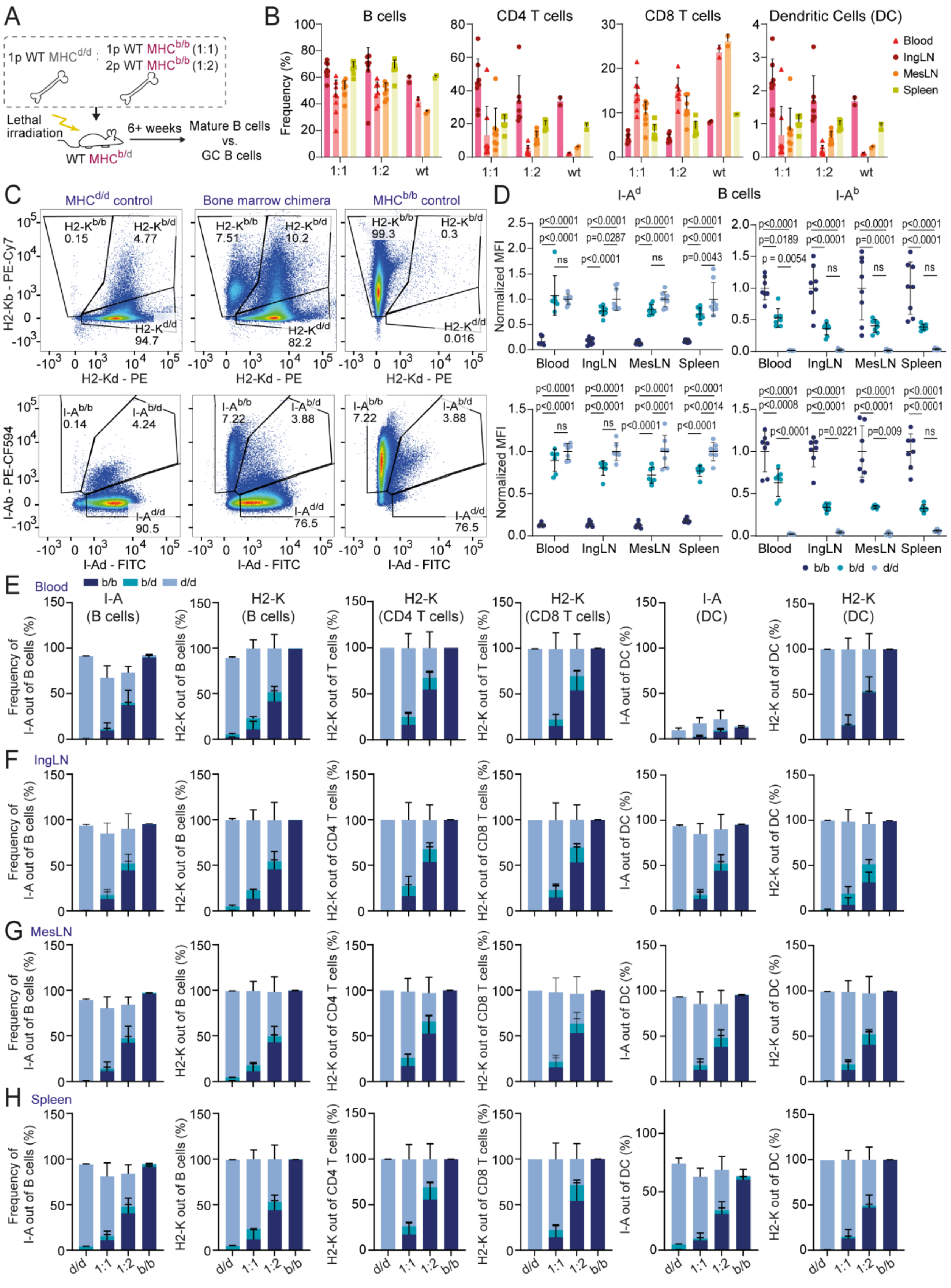

A
1p WT MHC d/d : 1p WT MHC b/b (1:1)
2p WT MHC b/b (1:2)
Lethal irradiation
WT MHC b/d
6+ weeks
Mature B cells vs. GC B cells
B
B cells
CD4 T cells
CD8 T cells
Dendritic Cells (DC)
Frequency (%)
Blood
IngLN
MesLN
Spleen
C
MHC d/d control
Bone marrow chimera
MHC b/b control
H2-Kb - PE-Cy7
H2-Kd - PE
I-Ab - PE-CF594
I-Ad - FITC
D
I-A d
B cells
I-A b
Normalized MFI
b/b
b/d
d/d
E
Blood
I-A (B cells)
H2-K (B cells)
H2-K (CD4 T cells)
H2-K (CD8 T cells)
I-A (DC)
H2-K (DC)
Frequency of I-A out of B cells (%)
F
IngLN
G
MesLN
H
Spleen

Supplementary Figure 5. The ability of I-$A^{d/d}$ B cells to dominate the repertoire of mixed chimeras is intrinsic. (A) Schematic overview showing the two groups of BM chimeras, consisting of lethally irradiated $MHC^{b/d}$ recipients, receiving one part (1p) $MHC^{d/d}$ and either one (1:1) or two (1:2) parts (2p) $MHC^{b/b}$ BM. (B) Frequencies of B cells, CD4 T cells, CD8 T cells, or dendritic cells (DC) in blood, inguinal and mesenteric lymph nodes (IngLN and MesLN), or spleen 8 weeks after reconstitution of 1:1 and 1:2 BM chimeras, and in C57BL/6J ($H2^{b/b}$) controls (wt). (C) Bivariate plots showing gating for H2-K^b vs. H2-K^d (top row) and I-A^b vs. I-A^d (bottom row) among splenic B cells of representative d/d controls (left), mixed BM chimeras (center) and b/b controls (right). (D) Normalized median fluorescence intensity of I-A^d (left) or I-A^b (right) for B cells of 1:1 (top) or 1:2 (bottom) chimeras, within subset expressing I-$A^{b/b}$, I-$A^{b/d}$ or I-$A^{d/d}$. Values were normalized to the signal for I-A^d in $MHC^{d/d}$ B cells (left) and I-A^b in $MHC^{b/b}$ B cells (right), respectively. (E) Haplotype ratios for I-A (left) or H2-K (second from left) among B cells, H2-K among CD4 (third from left) or CD8 (third from right) T cells, and I-A (second from right) or H2-K (right) among dendritic cells (DC), in blood of d/d and b/b controls as well as 1:1 and 1:2 mixed chimeras at 6 weeks post reconstitution. (F) As E, but for IngLN. (G) As E, but for MesLN. (H) As E, but for spleen. Bars and error bars represent mean±SD, n=8 (1:1 chim), 7 (1:2 chim), or 2 ($H2^{b/b}$ and $H2^{d/d}$ controls) mice per group. Statistical significance given for two-way ANOVA with Bonferroni's post-test. ns = $p>0.05$.

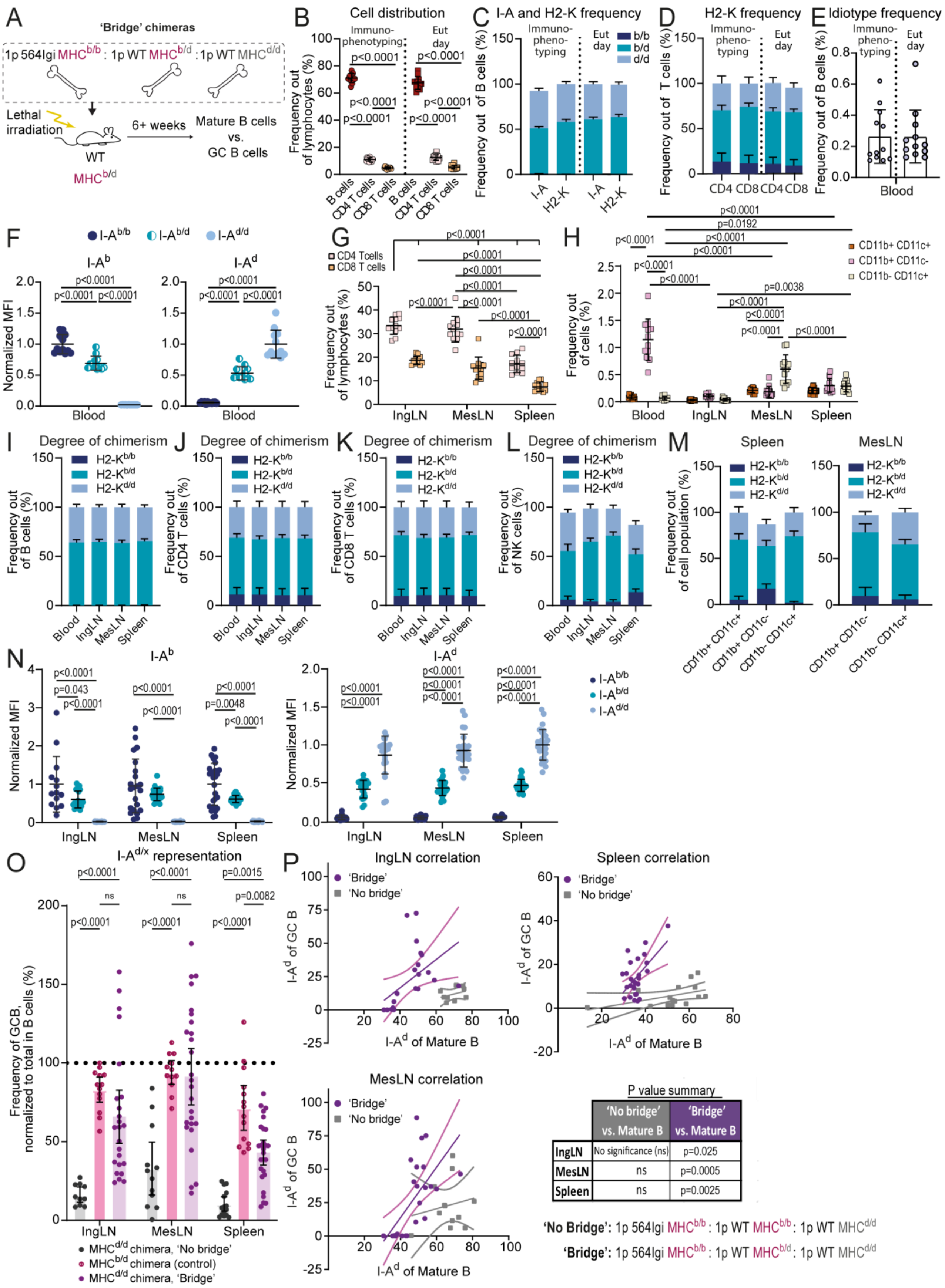

	'No bridge' vs. Mature B	'Bridge' vs. Mature B
IngLN	No significance (ns)	p=0.025
MesLN	ns	p=0.0005
Spleen	ns	p=0.0025

Supplementary Figure 6. Supporting data for main Figure 5. (A) Schematic overview showing the BM chimera setup. 1p = 1 part. (B) Distribution of B, CD4 T and CD8 T cells in blood of bridge chimeras at 6 (immunophenotyping) and at 7-9 weeks (euthanasia day, eut day) post reconstitution. (C) B cell I-A and H2-K haplotype frequencies in blood at 6 and 7-9 weeks. (D) H2-K haplotype frequencies of CD4 and CD8 T cells in blood at 6 and 7-9 weeks. (E) Idiotype frequencies at immunophenotyping and euthanasia day. (F) Normalized median fluorescence intensity (MFI) of I-A^b (left) and I-A^d (right) for B cells in blood at immunophenotyping. (G) CD4 and CD8 T cell frequencies at euthanasia. (H) CD11b+CD11c+, CD11b+CD11c- and CD11b-CD11c+ cell frequencies at euthanasia. H2-K haplotype distribution among B cells (I), CD4 T (J), CD8 T (K) and NK cells (L) in blood, IngLN, MesLN and spleen. (M) H2-K haplotype distribution among CD11b+CD11c+, CD11b+CD11c- and CD11b-CD11c+ cells from spleen (left) and MesLN (right). The number of CD11b+CD11c+ cells in MesLN was too low to determine the haplotype distribution within this subset. (N) Normalized MFI for I-A^b (left) and I-A^d (right), within I-$A^{b/b}$, I-$A^{b/d}$ and I-$A^{d/d}$ GC B cell compartments of chimeras, across IngLN, MesLN and spleen. (O) Representation of I-A^d cells within GCs relative to their representation in the mature B cell compartment, across IngLN, MesLN and spleen in the three chimera groups. The dashed line through 100% indicates 1:1 representation. (P) Correlation analysis of the frequency of I-A^d cells within the GC B population and the frequency of I-A^d within the mature B cell pool, for bridge and no bridge chimeras. In A-N, error bars represent mean±SD, with n=12-27 mice, from one or two independent experiments. In F and N, values were normalized to the signal for I-A^b in $MHC^{b/b}$ cells (left) and I-A^d in $MHC^{d/d}$ cells (right), respectively. Statistical tests used in B, G: two-way ANOVA with Šidák's post-test, in E: unpaired, two-tailed t-test with Welch's correction, in F: Kruskal-Wallis test with Dunn's post-test, in H, N, O: two-way ANOVA with Tukey's post-test. ns: $p>0.05$.

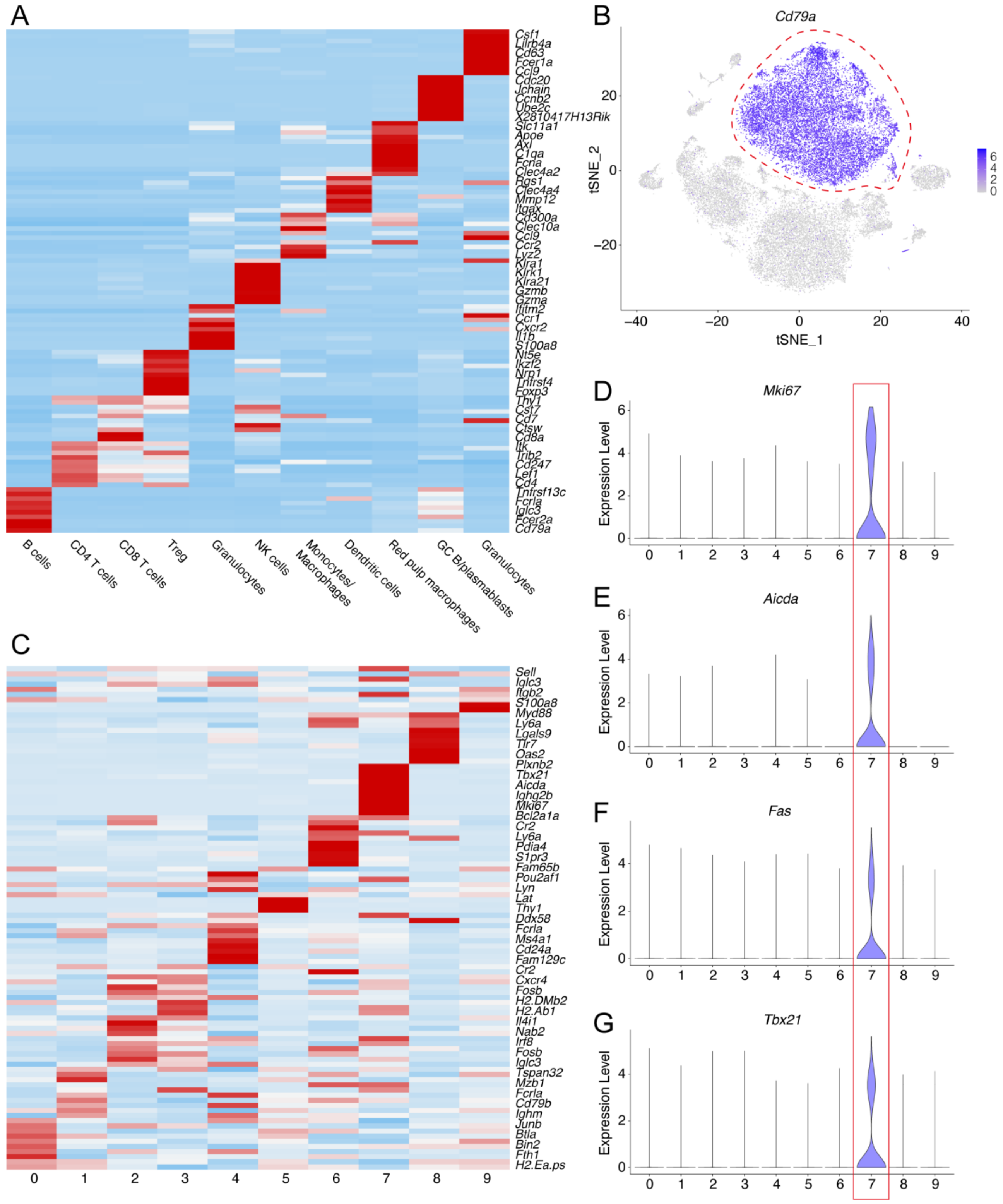

Supplementary Figure 7. Supporting data for main Figure 6. (A) Heatmap with the top 10 markers defining the clusters indicated in main Figure 6C. (B) Featureplot of *Cd79a* on the tSNE dimensionality reduction displayed in main Figure 6C. (C) Heatmap with the top 10 markers defining the clusters indicated in main Figure 6D. (D) Violin plot showing the expression of *Mki67* across the 10 clusters represented in panel C and main Figure 6D. (E) As D but showing expression of *Aicda*. (F) As D but showing expression of *Fas* (CD95). (G) As D but showing expression of *Tbx21* (T-bet). For C-G the clusters are defined as in Figure 6E.

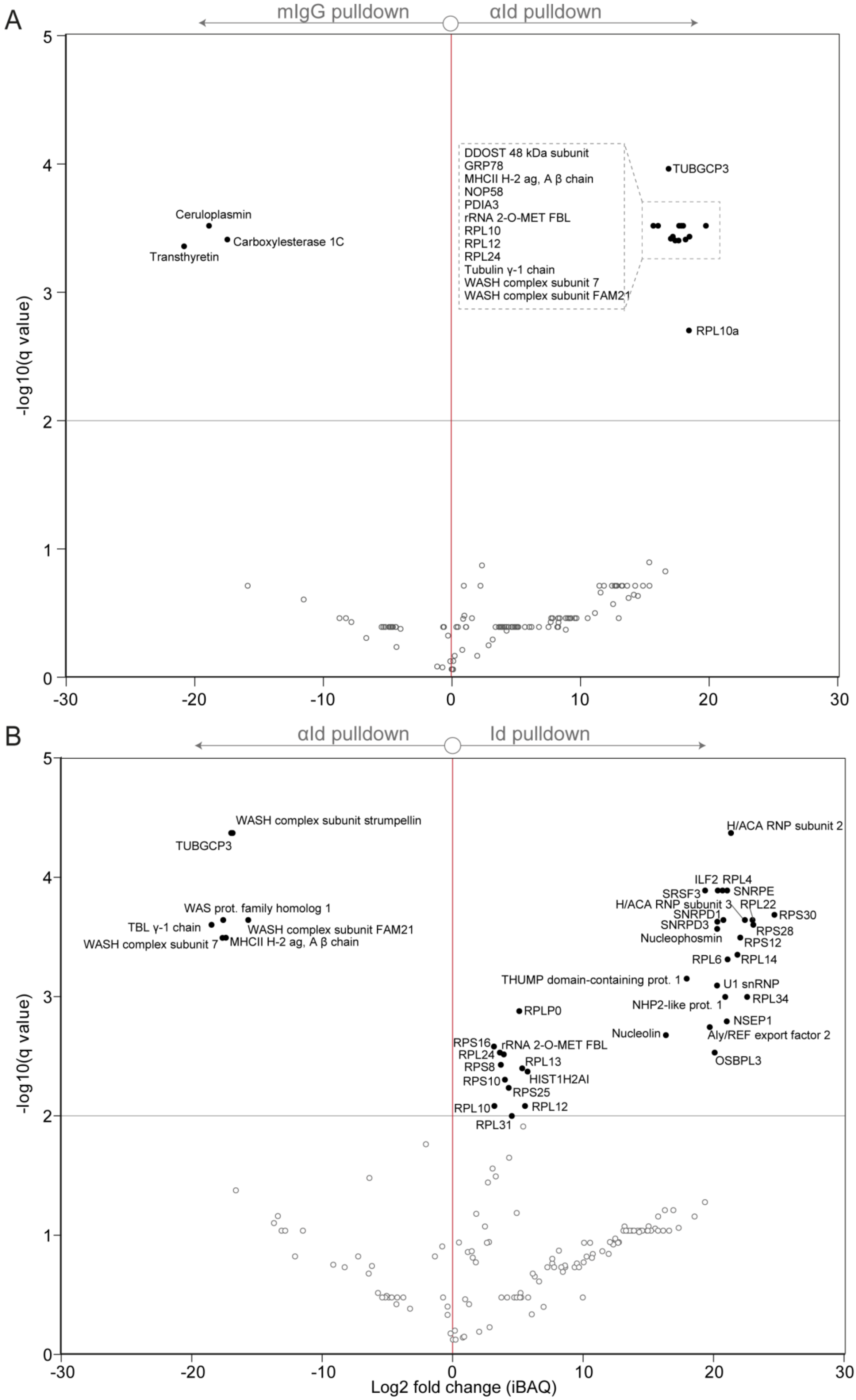
A
mIgG pulldown
αId pulldown
-log10(q value)
DDOST 48 kDa subunit
GRP78
MHCII H-2 ag, A β chain
NOP58
PDIA3
rRNA 2-O-MET FBL
RPL10
RPL12
RPL24
Tubulin γ-1 chain
WASH complex subunit 7
WASH complex subunit FAM21
TUBGCP3
RPL10a
Ceruloplasmin
Carboxylesterase 1C
Transthyretin
B
αId pulldown
Id pulldown
-log10(q value)
WASH complex subunit strumpellin
TUBGCP3
WAS prot. family homolog 1
TBL γ-1 chain
WASH complex subunit FAM21
WASH complex subunit 7
MHCII H-2 ag, A β chain
H/ACA RNP subunit 2
ILF2 RPL4
SRSF3
SNRPE
H/ACA RNP subunit 3
RPL22
SNRPD1
RPS30
SNRPD3
RPS28
Nucleophosmin
RPS12
RPL6
RPL14
THUMP domain-containing prot. 1
U1 snRNP
RPL34
NHP2-like prot. 1
RPLP0
NSEP1
Nucleolin
Aly/REF export factor 2
RPS16
rRNA 2-O-MET FBL
RPL24
OSBPL3
RPS8
RPL13
HIST1H2AI
RPS10
RPS25
RPL10
RPL12
RPL31
Log2 fold change (iBAQ)

Supplementary Figure 8. Supporting data for main Figure 7. (A) Volcano plot of mean differences in log2[iBAQ values] of clone 564 9D11 pulldowns (n=4) versus serum IgG pulldowns (n=4). (B) as A, but representing clone 564 C11 pulldowns (n=4) versus clone 564 9D11 pulldowns (n=4). The line at -log10(q value) = 2 indicates the significance threshold for paired t-test at p=0.01.

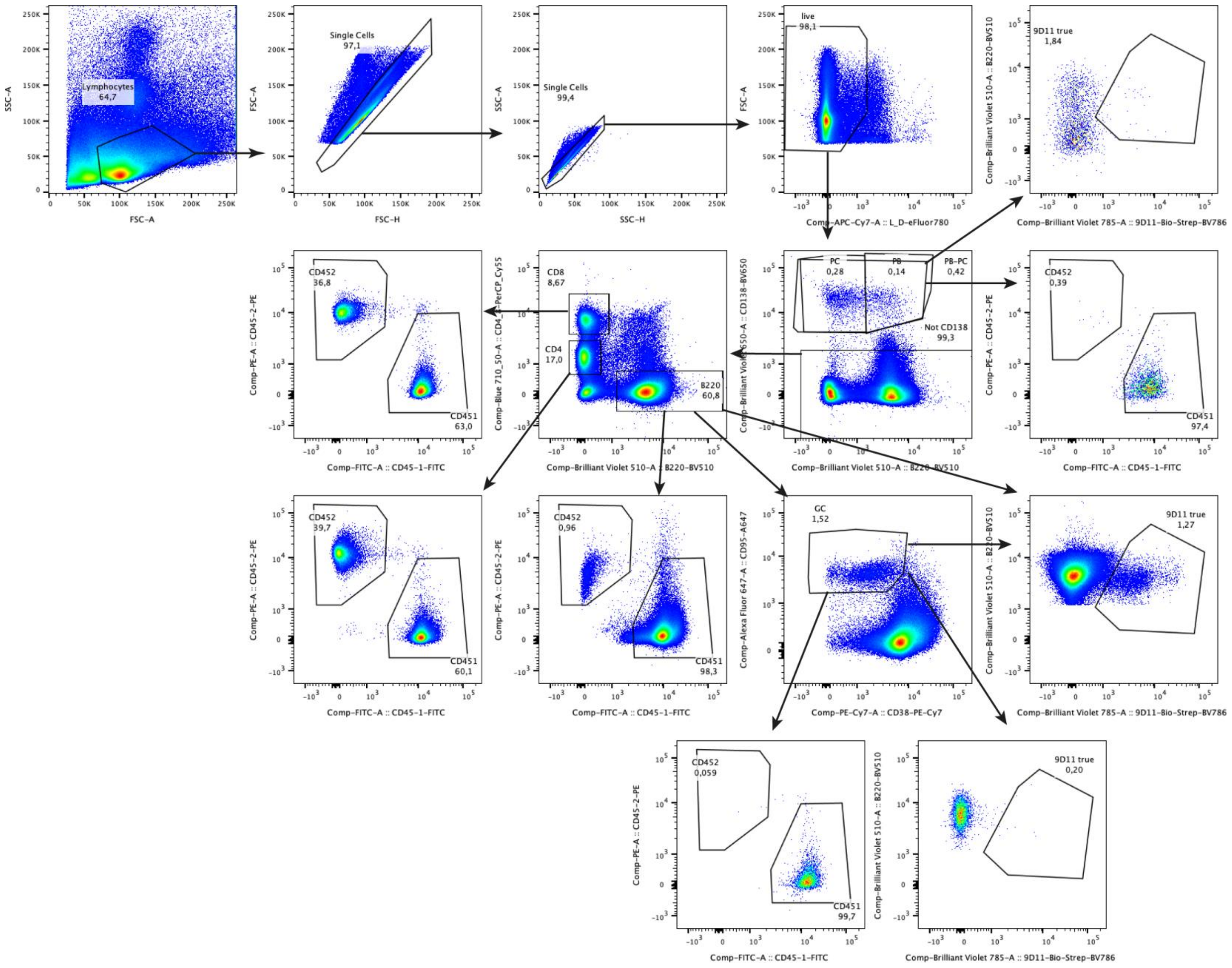
Lymphocytes
64,7
Single Cells
97,1
Single Cells
99,4
live
98,1
9D11 true
1,84
PC
0,28
PB
0,14
PB-PC
0,42
Not CD138
99,3
CD8
8,67
CD4
17,0
B220
60,8
CD452
36,8
CD451
63,0
CD452
0,39
CD451
97,4
CD452
39,7
CD451
60,1
CD452
0,96
CD451
98,3
GC
1,52
9D11 true
1,27
CD452
0,059
CD451
99,7
9D11 true
0,20
FSC-A
SSC-A
FSC-H
SSC-H
Comp-APC-Cy7-A :: L_D-eFluor780
Comp-Brilliant Violet 510-A :: B220-BV510
Comp-Brilliant Violet 785-A :: 9D11-Bio-Strep-BV786
Comp-FITC-A :: CD45-1-FITC
Comp-PE-A :: CD45-2-PE
Comp-PE-Cy7-A :: CD38-PE-Cy7
Comp-Alexa Fluor 647-A :: CD95-A647

Supplementary Figure 9: Representative gating strategy for Figure 1, 2, 3, S1, S2. Representative gating for B cells and GC B cells, CD4 and CD8 T cells, Idiotype positive (Id+) cells, plasmablasts (PB) and plasma cells (PC), as well as CD45.1 vs. CD45.2 subsets. Cells were gated by a lymphocyte gate, followed by two-fold exclusion of doublets, then exclusion of dead cells. Live singlets were gated as plasmablasts/cells (CD138+ PB-PC) or 'not CD138 positive', which were subgated as B cells (B220+), CD4 T cells (CD4+), and CD8 T cells (CD8+). B cells were further subgated into germinal center B cells (GC B). All subsets were gated as CD45.1+ vs. CD45.2+. For plasmablasts/cells, B cells and GC B cells, they were furthermore subgated for Idiotype+ cells (9D11+).

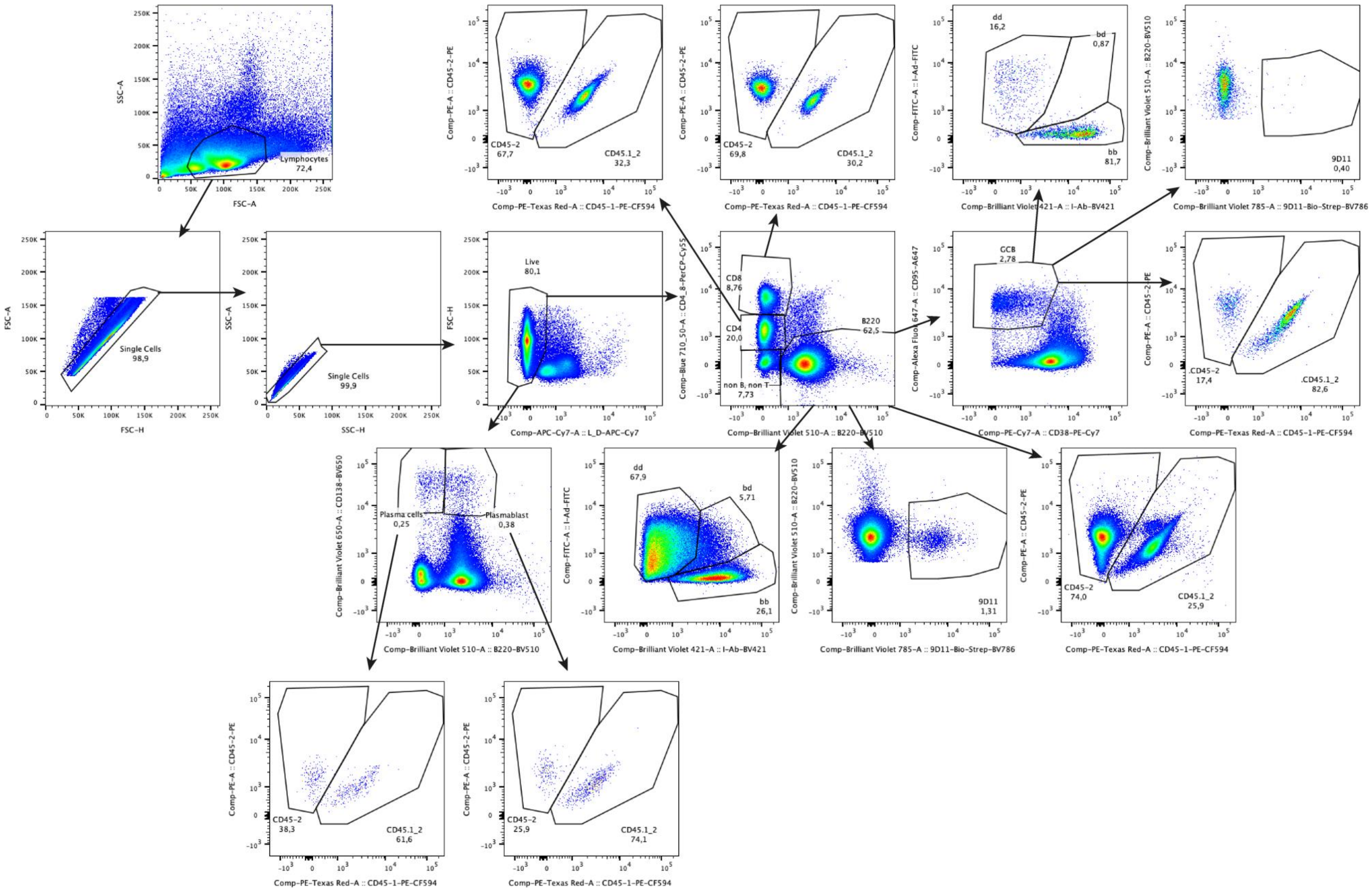
Lymphocytes
72,4
Single Cells
98,9
Single Cells
99,9
Live
80,1
CD45-2
67,7
CD45.1_2
32,3
CD45-2
69,8
CD45.1_2
30,2
dd
16,2
bd
0,87
bb
81,7
9D11
0,40
CD8
8,76
CD4
20,0
B220
62,5
non B, non T
7,73
GCB
2,78
CD45-2
17,4
CD45.1_2
82,6
Plasma cells
0,25
Plasmablast
0,38
dd
67,9
bd
5,71
bb
26,1
9D11
1,31
CD45-2
74,0
CD45.1_2
25,9
CD45-2
38,3
CD45.1_2
61,6
CD45-2
25,9
CD45.1_2
74,1
SSC-A
FSC-A
FSC-H
SSC-H
Comp-APC-Cy7-A :: L_D-APC-Cy7
Comp-PE-A :: CD45-2-PE
Comp-PE-Texas Red-A :: CD45-1-PE-CF594
Comp-FITC-A :: I-Ad-FITC
Comp-Brilliant Violet 421-A :: I-Ab-BV421
Comp-Brilliant Violet 510-A :: B220-BV510
Comp-Brilliant Violet 785-A :: 9D11-Bio-Strep-BV786
Comp-Blue 710_50-A :: CD4_8-PerCP-Cy55
Comp-Alexa Fluor 647-A :: CD95-A647
Comp-PE-Cy7-A :: CD38-PE-Cy7
Comp-Brilliant Violet 650-A :: CD138-BV650

Supplementary Figure 10: Representative gating strategy for Figure 4, 5, S4. Representative gating for B cells and GC B cells, CD4 and CD8 T cells, Idiotype positive (Id+) cells, plasmablasts and plasma cells, as well as CD45.1 vs. CD45.2 subsets along with I-Ab and I-Ad subsets. Cells were gated by a lymphocyte gate, followed by two-fold exclusion of doublets, then exclusion of dead cells. Live singlets were gated as plasmablasts or plasma cells, or subgated as B cells (B220+), CD4 T cells (CD4+), and CD8 T cells (CD8+). B cells were further subgated into germinal center B cells (GC B). All subsets were gated as CD45.1+ vs. CD45.2+. For B cells and GC B cells, they were furthermore subgated for Idiotype+ cells (9D11+) or subgated as I-Ab/b, I-Ab/d or I-Ad/d.

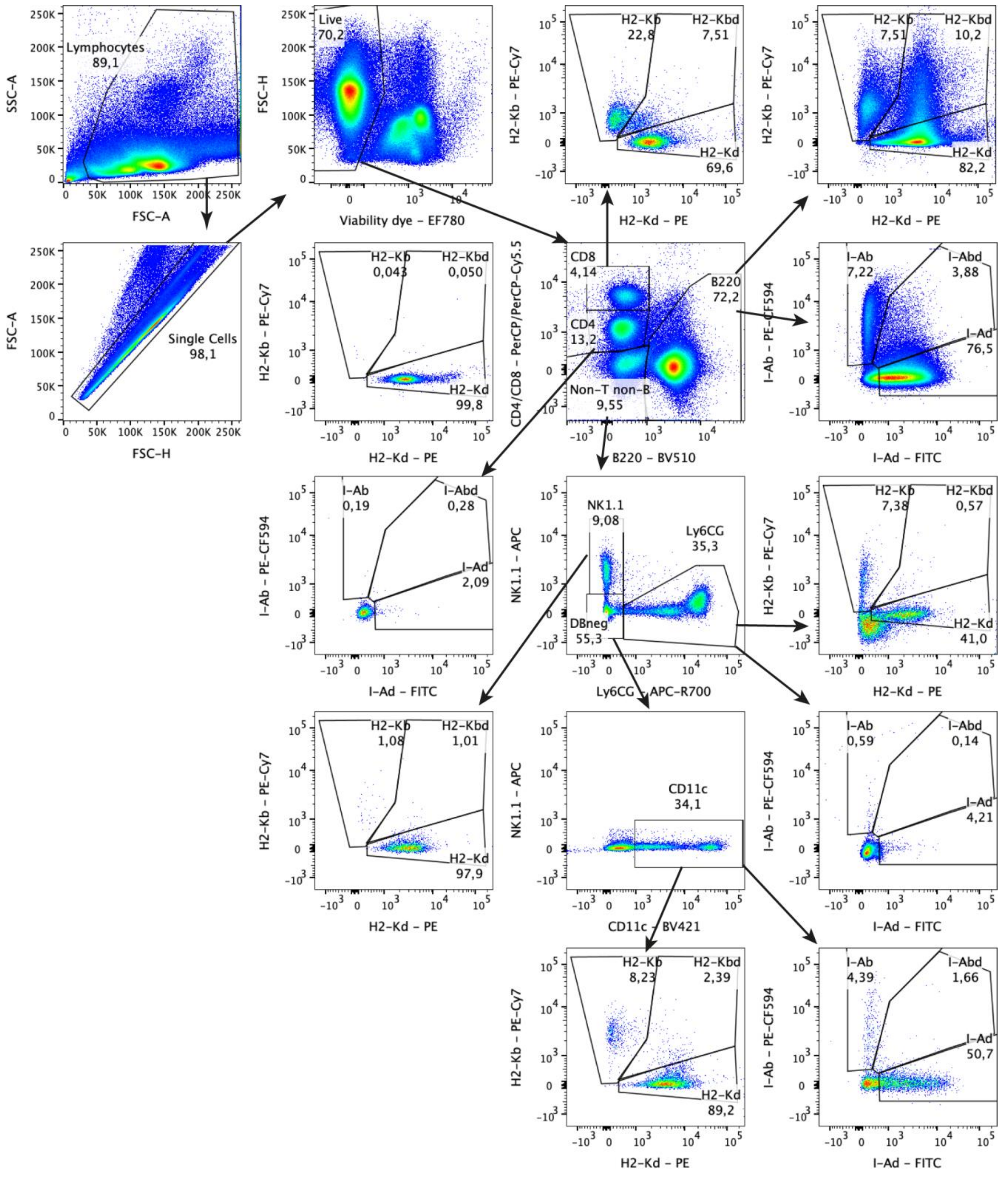

Supplementary Figure 11: Representative gating strategy for Supplementary Figure 5. Cells were gated generously by a lymphocyte (leukocyte) gate, followed by exclusion of doublets, then exclusion of dead cells. Live singlets were gated as B cells (B220+), CD4 T cells (CD4+), CD8 T cells (CD8+) or non-T non-B cells, which were further subdivided into monocytes/granulocytes (Ly6CG+), NK cells (NK1.1), and double negatives (DBneg), which were again further gated for dendritic cells (CD11c+). All subsets were subdivided into H2-Kb, H2-Kd, and H2Kb/d, and B cells, monocytes/granulocytes, and dendritic cells were additionally subdivided into I-Ab, I-Ad and I-Ab/d.

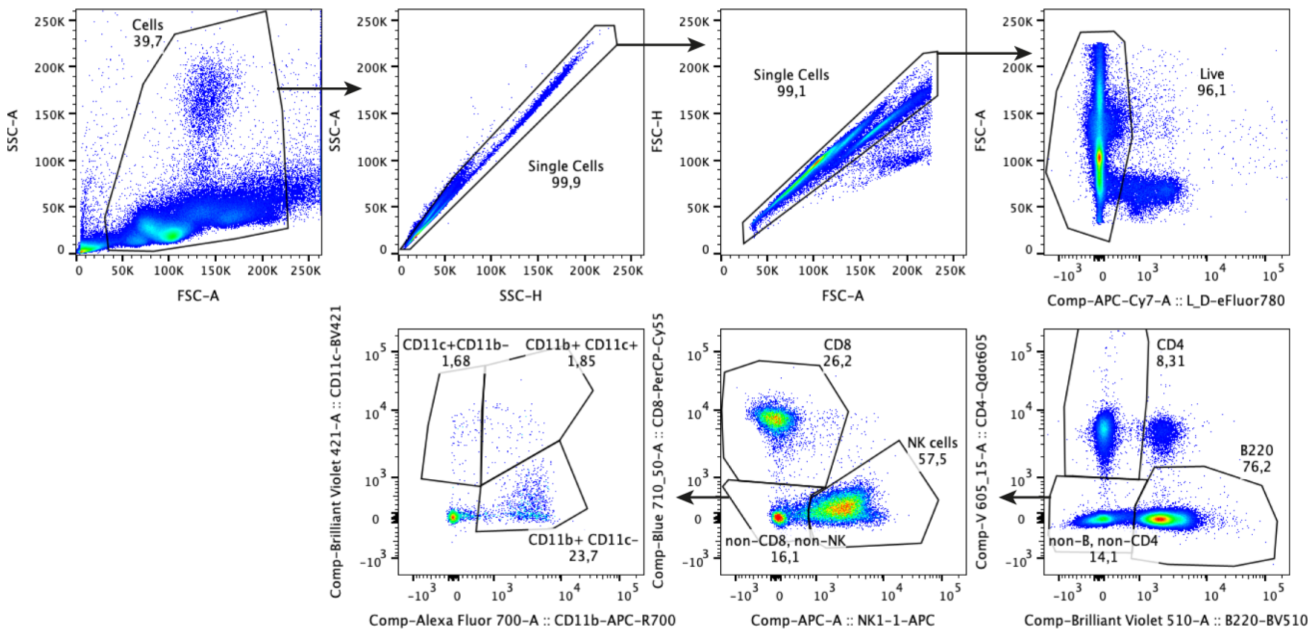

Supplementary Figure 12: Representative gating strategy for Supplementary Figure 6. A generous cell gate was followed by two-fold exclusion of doublets, then exclusion of dead cells. Live singlets were gated as B cells (B220+), CD4 T cells (CD4+), or non-B, non-CD4 cells. The latter were further subdivided into NK cells (NK1.1), CD8 T cells, and non-CD8, non-NK, which were again further subgated as CD11b+CD11c-, CD11b+CD11c+, and CD11b-CD11c+.

Supplementary Tables

	Supplementary Table 1			
**Antibody**	**Clone**	**Source**	**Identifier**	**Dilution**
Anti-Idiotype-A647	564-9D11	Hybridoma kindly provided by Elisabeth Alicot, Boston Children's Hospital	ref. Chatterjee et al., Eur J Immunol. 2013 Sep;43(9):2441-2450. doi: 10.1002/eji.201343412	1/500
Anti-Idiotype-biotin	564-9D11	Hybridoma kindly provided by Elisabeth Alicot, Boston Children's Hospital	ref. Chatterjee et al., Eur J Immunol. 2013 Sep;43(9):2441-2450. doi: 10.1002/eji.201343412	1/500
Anti-B220-A647	RA3-6B2	BD Biosciences	Cat# 557683	1/500
Anti-B220-V500	RA3-6B2	BD Biosciences	Cat# 561227	1/500
Anti-B220-A700	RA3-6B2	BD Biosciences	Cat# 557957	1/500
Anti-B220-BV510	RA3-6B2	BD Horizon	Cat# 563103	1/500
Anti-B220-BV650	RA3-6B2	BD Horizon	Cat# 563893	1/500
Anti-B220-PB	RA3-6B2	BD Pharmingen	Cat# 558108	1/500
Anti-CD11b-APC-A700	M1/70	BD Pharmingen	Cat# 564985	1/500
Anti-CD11c-BV421	N418	BD Pharmingen	Cat# 565451	1/500
Anti-CD138-BV650	281-2	BD	Cat# 564068	1/500
Anti-CD38-PE-Cy7	90	BioLegend	Cat# 102718	1/500
Anti-CD45.1-FITC	A20	BioLegend	Cat# 110706	1/500
Anti-CD45.1-PE-CF594	A20	BD Horizon	Cat# 562452	1/500
Anti-CD45.1-E450	A20	Life Technologies	Cat# 48-0453-82	1/500
Anti-CD45.2-AF594	104	BioLegend	Cat# 109850	1/500
Anti-CD45.2-APC	104	BioLegend	Cat# 109814	1/500
Anti-CD45.2-BV786	104	BD Horizon	Cat# 563686	1/500
Anti-CD45.2-PE	104	BioLegend	Cat# 109808	1/500
Anti-CD4-PerCP	RM4-5	BioLegend	Cat# 100538	1/300
Anti-CD4-qDot605	RM4-5	ThermoFisher Scientific	Cat# Q10092	1/500
Anti-CD8-PerCP-Cy5.5	SK1	BD Pharmingen	Cat# 565310	1/300
Anti-CD95-unlabelled -> iFluor647	Jo2	BD Pharmingen -> In-house labeled	Cat# 554254	1/500
Anti-CD95-PE	DX2	BD Pharmingen	Cat# 561976	1/500
eBioscience™ Fixable Viability Dye - eFlour780	n/a	Thermo Fisher Scientific	Cat# 65–0865-14	1/2,000
Anti-H2kb-PE-Cy7	AF6-88.5	BioLegend	Cat# 116520	1/500
Anti-H2kd-PE	SF1-1.1	BioLegend	Cat# 116608	1/500
Anti-I-Ab-BV421	AF6-120.1	BD Horizon	Cat# 562928	1/500
Anti-I-Ab-PE	AF6-120.1	BioLegend	Cat# 116408	1/500
Anti-I-Ab-PE-CF594	AF6-120.1	BD	Cat# 562824	1/500
Anti-I-Ad-FITC	39-10-8	BD Pharmingen	Cat# 553610	1/500
Anti-I-Ad-AF488	39-10-8	BioLegend	Cat# 115008	1/500

AF488 anti-mouse IgD	11-26c.2a	BioLegend	Cat# 405718	1/500
Anti-Ki67-eflour660	SolA15	ThermoFisher	Cat# 50-5698-82	1/500
IgD-PB	11-26c.2a	BioLegend	Cat# 405712	1/500
Streptavidin-PE	n/a	BioLegend	Cat# 405204	1/500
Streptavidin-BV785	n/a	BD Biosciences	Cat# 563858	1/500
Biotin anti-mouse NK1.1 Antibody	PK136	BioLegend	Cat# 108704	1/500
Biotin anti-mouse CD3epsilon Antibody	145-2C11	BioLegend	Cat# 100304	1/500
Biotin anti-mouse CD8a Antibody	53-6-7	BioLegend	Cat# 100704	1/500
Anti-Ly6g/c-APC-R700	RB6-8C5	BD Pharmingen	Cat# 565510	1/500
Biotin rat anti-mouse CD4 Antibody	RM4-5	BD Pharmingen	Cat# 553045	1/500
Biotin anti-mouse TER-119/ Erythroid Cells Antibody	TER-119	Nordic Biosite	Cat# 116204	1/500
Biotinylated goat-anti-mouse Ig	Polyclonal	Southern Biotech	Cat# 1010-08	1/500
Anti-biotin microbeads	n/a	Miltenyi Biotec	Cat# 130-090-485	n/a
PNA microbead kit	n/a	Miltenyi Biotec	Cat# 130-110-479	n/a
Anti-NK1.1-APC	2F1	BD Biosciences	Cat# 561117	1/500
Biotin anti-mouse TCR beta chain Antibody	H57-597	Nordic Biosite	Cat# 109204	1/500
Fc-block (anti-CD16/32)	2.4G2	BD	Cat# 553142	1/300
Idiotype antibody-biotin	564-C11	Hybridoma kindly provided by Elisabeth Alicot, Boston Children's Hospital	ref. Chatterjee et al., Eur J Immunol. 2013 Sep;43(9):2441-2450. doi: 10.1002/eji.201343412	1/500

Supplementary Table 2. Markers used for gating various cell types. All cells are previously gated as lymphocytes (based on FSC-A vs SSC-A plot), single cells (based on FSC-A vs FSC-H and SSC-A vs SSC-H), and live (based on being negative for the Fixable Viability Dye).

**Subset**	**Markers**
B cell	B220 positive
CD4 T cell	CD4 positive
CD8 T cell	CD8 positive
GC B cell	B220 positive, CD38 low and CD95 positive
Plasmablasts (PB)	CD138 positive, B220 high
Plasma cells (PC)	CD138 positive, B220 low
PB/PC	CD138 positive, B220 positive
NK cells	CD4, B220 and CD8 negative. NK1.1 positive
Ly6g/c cells	CD4, B220 and CD8 negative. Ly6g/c positive
Dendritic cells	CD4, B220 and CD8 negative. NK1.1 and Ly6g/c negative. CD11c positive
