# Supplementary material for: Antigen presentation by B cells enables epitope spreading across an MHC barrier
Source: Nat Commun. 2023 Oct 31;14:6941. doi: 10.1038/s41467-023-42541-7 (PMC10618542; doi:10.1038/s41467-023-42541-7)
Supplement: Supplementary file 3 — Reporting Summary [file 41467_2023_42541_MOESM3_ESM.pdf]

nature portfolio

Corresponding author(s): Søren E. Degn

Last updated by author(s): Oct 5, 2023

Reporting Summary

Nature Portfolio wishes to improve the reproducibility of the work that we publish. This form provides structure for consistency and transparency in reporting. For further information on Nature Portfolio policies, see our Editorial Policies and the Editorial Policy Checklist.

Statistics

For all statistical analyses, confirm that the following items are present in the figure legend, table legend, main text, or Methods section.

n/a	Confirmed	
☐	☒	The exact sample size (n) for each experimental group/condition, given as a discrete number and unit of measurement
☐	☒	A statement on whether measurements were taken from distinct samples or whether the same sample was measured repeatedly
☐	☒	The statistical test(s) used AND whether they are one- or two-sided<br>*Only common tests should be described solely by name; describe more complex techniques in the Methods section.*
☒	☐	A description of all covariates tested
☐	☒	A description of any assumptions or corrections, such as tests of normality and adjustment for multiple comparisons
☐	☒	A full description of the statistical parameters including central tendency (e.g. means) or other basic estimates (e.g. regression coefficient) AND variation (e.g. standard deviation) or associated estimates of uncertainty (e.g. confidence intervals)
☐	☒	For null hypothesis testing, the test statistic (e.g. F, t, r) with confidence intervals, effect sizes, degrees of freedom and P value noted<br>*Give P values as exact values whenever suitable.*
☒	☐	For Bayesian analysis, information on the choice of priors and Markov chain Monte Carlo settings
☒	☐	For hierarchical and complex designs, identification of the appropriate level for tests and full reporting of outcomes
☒	☐	Estimates of effect sizes (e.g. Cohen's d, Pearson's r), indicating how they were calculated

Our web collection on statistics for biologists contains articles on many of the points above.

Software and code

Policy information about availability of computer code

Data collection	Agilent NovoExpress v. 1.5.0<br>BD FACSDiva v. 8.0.2<br>ZEN v. 2 (Blue edition)<br>VS-ASW v. 3.4.1
Data analysis	Microsoft Excel v. 16.77.1<br>FlowJO version 10.8.1 with UMAP v. 3.1<br>FCS Express v. 7<br>GraphPad Prism v. 9<br>OlyVIA Image Viewer v. 3.12<br>BD Rhapsody Targeted Analysis Pipeline in SevenBridges, CWL Version v1.2, v1.0<br>SeqGeq 1.7.0<br>R v. 4.1.2 using Seurat v. 4.0.6, DoubletFindr v. 2.0.3, FactoMineR v. 2.8<br>ImageJ version 1.52P (Fiji v. 2.1.0/1.53c)<br>MaxQuant v. 1.5.3.30

For manuscripts utilizing custom algorithms or software that are central to the research but not yet described in published literature, software must be made available to editors and reviewers. We strongly encourage code deposition in a community repository (e.g. GitHub). See the Nature Portfolio guidelines for submitting code & software for further information.

Data

Policy information about availability of data

All manuscripts must include a data availability statement. This statement should provide the following information, where applicable:

- Accession codes, unique identifiers, or web links for publicly available datasets
- A description of any restrictions on data availability
- For clinical datasets or third party data, please ensure that the statement adheres to our policy

The data that support this study are available from the corresponding author upon request.
Processed data and raw data for single cell RNA sequencing produced in this study are available via the Gene Expression Omnibus (GSE202358) (https://www.ncbi.nlm.nih.gov/geo/query/acc.cgi?acc=GSE202358). We have furthermore established easily accessible databases for the total dataset: (https://dreamapp.biomed.au.dk/B_cells_inclusion_TOTAL/), and B cell dataset: (https://dreamapp.biomed.au.dk/B_cells_inclusion_B_cells/)
The mass spectrometry proteomics data have been deposited to the ProteomeXchange Consortium via the PRIDE71 partner repository with the dataset identifier PXD044323 (http://www.ebi.ac.uk/pride/archive/projects/PXD044323). Raw data for the autoantigen array screenings have been provided with Source Data and additional raw data shown in the figures are provided in the Source Data file.

Human research participants

Policy information about studies involving human research participants and Sex and Gender in Research.

Reporting on sex and gender	No human subjects were involved in the present study.
Population characteristics	No human subjects were involved in the present study.
Recruitment	No human subjects were involved in the present study.
Ethics oversight	No human subjects were involved in the present study.

Note that full information on the approval of the study protocol must also be provided in the manuscript.

Field-specific reporting

Please select the one below that is the best fit for your research. If you are not sure, read the appropriate sections before making your selection.

☒ Life sciences ☐ Behavioural & social sciences ☐ Ecological, evolutionary & environmental sciences

For a reference copy of the document with all sections, see nature.com/documents/nr-reporting-summary-flat.pdf

Life sciences study design

All studies must disclose on these points even when the disclosure is negative.

Sample size	Sample size calculations were not performed a priori. Group sizes were chosen based on the typical number of replicates reported for similar studies as outlined in Festing MFW, "On determining sample size in experiments involving laboratory animals", 2018 Aug;52(4):341-350. doi: 10.1177/0023677217738268, relying on group sizes of Green et al., Front Immunol. 2021 Dec 6;12:782558. doi: 10.3389/fimmu.2021.782558; Voss et al., Front Immunol. 2022 Dec 14;13:1021370. doi: 10.3389/fimmu.2022.1021370; and Degn et al., Cell. 2017 Aug 24;170(5):913-926.e19. doi: 10.1016/j.cell.2017.07.026; for similar experimental setups and read-outs.
Data exclusions	For four-way chimeras (3 donors into 1 recipient), a few mice were excluded where a highly skewed donor compartment ratio was observed, i.e., where any one of the wild-type (non-564Igi) compartments constituted less than 1/6 of total.
Replication	All experiments were repeated three times independently unless otherwise noted. All attempts at replication were successful.
Randomization	Randomization was not performed in this study because inbred transgenic animals and chimeras derived hereof were used throughout. Hence, experimental group assignment was determined directly by the appropriate genotype or bone marrow composition for each group.
Blinding	No blinding was performed. Sample identity was inherently represented by the read-outs, precluding blinding during gating. However, universal gating was employed for each experiment. All read-outs were quantitative, rather than qualitative, minimizing the risk of experimenter bias in interpreting the results.

Reporting for specific materials, systems and methods

We require information from authors about some types of materials, experimental systems and methods used in many studies. Here, indicate whether each material, system or method listed is relevant to your study. If you are not sure if a list item applies to your research, read the appropriate section before selecting a response.

Materials & experimental systems

n/a	Involved in the study	
☐	☒	Antibodies
☒	☐	Eukaryotic cell lines
☒	☐	Palaeontology and archaeology
☐	☒	Animals and other organisms
☒	☐	Clinical data
☒	☐	Dual use research of concern

Methods

n/a	Involved in the study	
☒	☐	ChIP-seq
☐	☒	Flow cytometry
☒	☐	MRI-based neuroimaging

Antibodies

Antibodies used

Anti-9D11-A647 produced in-house from hybridoma kindly provided by Elisabeth Alicot, Boston Children's Hospital, ref. Chatterjee et al., Eur J Immunol. 2013 Sep;43(9):2441-2450. doi: 10.1002/eji.201343412
Anti-9D11-biotin produced in-house from hybridoma as above.
Anti-B220-A647 BD Biosciences Cat#557683, Clone RA3-6B2, Reactivity: Mouse (QC Testing), Human (Reported), Application: Flow cytometry (Routinely Tested), Immunofluorescence (Tested During Development).
Anti-B220-V500 BD Biosciences Cat#561227, Clone RA3-6B2, Reactivity: Mouse (QC Testing), Human (Reported), Application: Flow cytometry (Routinely Tested).
Anti-B220-A700 BD Biosciences Cat#557957, Clone RA3-6B2, Reactivity: Mouse (QC Testing), Human (Reactivity Confirmed in Development), Application: Flow cytometry (Routinely Tested).
Anti-B220-BV510 BD Horizon Cat#563103, Clone RA3-6B2, Reactivity: Mouse (QC Testing), Application: Flow cytometry (Routinely Tested).
Anti-B220-BV650 BD Horizon Cat#563893, Clone RA3-6B2, Reactivity: Mouse (QC Testing), Application: Flow cytometry (Routinely Tested).
Anti-B220-PB BD Pharmingen Cat#558108, Clone RA3-6B2, Reactivity: Mouse (QC Testing), Human (Reported), Application: Flow cytometry (Routinely Tested).
Anti-CD11b-APC-A700 BD Pharmingen Cat#564985, Clone M1/70, Reactivity: Mouse (QC Testing), Human (Tested in Development), Application: Flow cytometry (Routinely Tested).
Anti-CD11c-BV421 BD Pharmingen Cat#565451, Clone N418, Reactivity:Mouse (QC Testing), Application: Flow cytometry (Routinely Tested).
Anti-CD138-BV650 BD Cat#564068, Clone 281-2, Reactivity: Mouse (QC Testing), Application: Flow cytometry (Routinely Tested).
Anti-CD38-PE-Cy7 BioLegend Cat#102718, Clone 90, Verified Reactivity: Mouse (QC Testing), Application: FC - Quality tested.
Anti-CD45.1-FITC BioLegend Cat#110706, Clone A20, Verified Reactivity: Mouse (QC Testing), Application: FC - Quality tested.
Anti-CD45.1-PE-CF594 BD Horizon Cat#562452, Clone A20, Reactivity: Mouse (QC Testing), Application: Flow cytometry (Routinely Tested).
Anti-CD45.1-E450 Life Technologies Cat#48-0453-82, Clone A20, Species Reactivity: Mouse (QC Testing), Application: Immunohistochemistry (Frozen) (IHC (F)), Flow Cytometry (Flow).
Anti-CD45.2-AF594 BioLegend Cat#109850, Clone 104, Verified Reactivity: Mouse (QC Testing), Application: IHC-F - Quality tested.
Anti-CD45.2-APC BioLegend Cat#109814, Clone 104, Verified Reactivity: Mouse (QC Testing), Application: FC - Quality tested.
Anti-CD45.2-BV786 BD Horizon Cat#563686, Clone 104, Reactivity: Mouse (QC Testing), Application: Flow cytometry (Routinely Tested).
Anti-CD45.2-PE BioLegend Cat#109808, Clone 104, Verified Reactivity: Mouse (QC Testing), Application: FC - Quality tested.
Anti-CD4-PerCP BioLegend Cat#100538, Clone RM4-5, Verified Reactivity: Mouse (QC Testing), Application: FC - Quality tested.
Anti-CD4-qDot605 ThermoFisher Scientific Cat#Q10092, Clone RM4-5, Species Reactivity: Mouse (QC Testing), Application: Flow Cytometry (Flow).
Anti-CD8-PerCP-Cy5.5 BD Pharmingen Cat#565310, Clone SK1, Reactivity: Human (QC Testing), Rhesus, Cynomolgus, Baboon (Tested in Development), Application: Flow cytometry (Routinely Tested).
Anti-CD95-unlabelled BD Pharmingen Cat#554254 (labeled in-house with iFluor647), Clone Jo2, Reactivity: Mouse (QC Testing), Application: Flow cytometry (Routinely Tested), Cytotoxicity, Immunoprecipitation (Reported).
Anti-CD95-PE BD Pharmingen Cat#561976, Clone DX2, Reactivity: Human (QC Testing), Rhesus, Cynomolgus, Baboon (Tested in Development), Application: Flow cytometry (Routinely Tested).
Anti-H2kb-PE-Cy7 BioLegend Cat#116520, Clone AF6-88.5, Verified Reactivity: Mouse (QC Testing), Application: FC - Quality tested.
Anti-H2kd-PE BioLegend Cat#116608, Clone SF1-1.1, Verified Reactivity: Mouse (QC Testing), Application: FC - Quality tested.
Anti-I-Ab-BV421 BD Horizon Cat#562928, Clone AF6-120.1, Reactivity: Mouse (QC Testing), Application: Flow cytometry (Routinely Tested).
Anti-I-Ab-PE BioLegend Cat#116408, Clone AF6-120.1, Verified Reactivity: Mouse (QC Testing), Application: FC - Quality tested.
Anti-I-Ab-PE-CF594 BD Cat#562824, Clone AF6-120.1, Reactivity: Mouse (QC Testing), Application: Flow cytometry (Routinely Tested).
Anti-I-Ad-FITC BD Pharmingen Cat#553610, Clone 39-10-8, Reactivity: Mouse (QC Testing), Application: Flow cytometry (Routinely Tested).
Anti-I-Ad-AF488 BioLegend Cat#115008, Clone 39-10-8, Verified Reactivity: Mouse (QC Testing), Application: FC - Quality tested.
Anti-mouse IgD-AF488 BioLegend Cat#405718, Clone 11-26c.2a, Verified Reactivity: Mouse (QC Testing), Application: FC - Quality tested, SB - Reported in the literature, not verified in house.
Anti-Ki67-eflour660 ThermoFisher Cat#50-5698-82, Clone SolA15, Species Reactivity: Dog, Cynomolgus monkey, Human, Mouse, Non-human primate, Rat, Applications: WB, IHC, Paraffin (IHC (P)), ICC/IF, Flow, FN.

	Anti-IgD-PB BioLegend Cat#405712, Clone 11-26c.2a, Verified Reactivity: Mouse (QC Testing), Application: FC - Quality tested.<br>Biotin anti-mouse NK1.1 Antibody BioLegend Cat#108704, Clone PK136, Verified Reactivity: Mouse (QC Testing), Application FC - Quality tested.<br>Biotin anti-mouse CD3epsilon Antibody BioLegend Cat#100304, Clone 145-2C11, Verified Reactivity: Mouse (QC Testing), Application FC - Quality tested, IHC-F - Verified.<br>Biotin anti-mouse CD8a Antibody BioLegend Cat#100704, 53-6.7, Verified Reactivity: Mouse (QC Testing), Application: FC - Quality tested, IHC - Reported in the literature, not verified in house.<br>Anti-Ly6g/c-APC-R700 BD Pharmingen Cat#565510, Clone RB6-8C5, Reactivity: Mouse (QC Testing), Application: Flow cytometry (Routinely Tested).<br>Biotin rat anti-mouse CD4 Antibody BD Pharmingen Cat#553045, Clone RM4-5, Reactivity: Mouse (QC Testing), Application: Flow cytometry (Routinely Tested).<br>Biotin anti-mouse TER-119/Erythroid Cells Antibody BioLegend Cat# 116204, Clone TER-119, Verified Reactivity: Mouse (QC Testing), Application: FC - Quality tested.<br>Biotinylated goat-anti-mouse Ig Southern Biotech Cat# 1010-08, polyclonal, Reactivity: Mouse (QC Testing), Application ELISA, Flow cytometry (tested).<br>Anti-NK1.1-APC BD Biosciences Cat#561117, Clone 2F1, Reactivity: Mouse (QC Testing), Application: Flow cytometry (Routinely Tested)-<br>Biotin anti-mouse TCR beta chain Antibody BioLegend Cat#109204, Clone H57-597, Verified Reactivity: Mouse (QC Testing), Application: FC - Quality tested, IHC-F - Verified.<br>Anti-CD16/32 (Fc-block) BD Cat#553142, Clone 2.4G2, Reactivity: Mouse (QC Testing), Application:<br>Blocking, Flow cytometry (Routinely Tested), Immunohistochemistry-frozen (Tested During Development), Immunoprecipitation (Reported).
Validation	Vendor validation throughout, as indicated above, except for 564-9D11 and 564-C11, which were obtained from Elisabeth Alicot (validated in Chatterjee et al., EJI 2013), and validated in-house by titration against idiotype 564-C11 and anti-idiotype 564-9D11, respectively, in a time-resolved immunfluorometric assay.

Animals and other research organisms

Policy information about studies involving animals; ARRIVE guidelines recommended for reporting animal research, and Sex and Gender in Research

Laboratory animals	B6.SJL-Ptprca Pepcb/BoyJ, Jackson Laboratories, RRID:IMSR_JAX:002014.<br>B6.Cg-Ptprca Tg(UBC-PA-GFP)1Mnz/J, Jackson Laboratories, RRID:IMSR_JAX:022486.<br>B6.129S2-H2dlAb1-Ea/J, Jackson Laboratories, RRID:IMSR_JAX:003584.<br>B6.129S4(Cg)-Igktm1(Igk564)Tik Ightm1(Igh564)Tik/J, made available by Thereza Imanishi-Kari, Tufts University, for the present study, and has since become available at Jackson Laboratories, RRID:IMSR_JAX:032723.<br>Tg(Aicda-hCD2-Cre)9Mbu (Aicda-Cre) were kindly provided by Meinrad Busslinger, Research Institute of Molecular Pathology, Vienna Biocenter.<br>B6.129(Cg)-FoxP3tm4(YFP/icre)Ayr/J, Jackson Laboratories, RRID:IMSR_JAX:016959.<br>B6.129S(FVB)-Bcl6tm1.1Dent/J, Jackson Laboratories, RRID:IMSR_JAX:023727.<br>C57BL/6JRj Mouse, Janvier Labs, RRID:IMSR_JAX:000664.<br>Both male and female mice were used in experiments. Bone marrow recipients were 8-18 weeks old at irradiation and donors were 7-22 weeks old at time of bone marrow harvest. The mice were maintained in our SPF vivarium at the Department of Biomedicine, Aarhus University, in individually ventilated cages on a standard 12-hr light/dark cycle, with standard chow and water ad libitum, at ambient room temperature (20-22 degrees C) and ambient humidity.
Wild animals	This study did not involve wild animals.
Reporting on sex	Both male and female mice were used in experiments.
Field-collected samples	This study did not involve samples collected from the field.
Ethics oversight	The Danish Animal Experiments Inspectorate.

Note that full information on the approval of the study protocol must also be provided in the manuscript.

Flow Cytometry

Plots

Confirm that:

- [x] The axis labels state the marker and fluorochrome used (e.g. CD4-FITC).
- [x] The axis scales are clearly visible. Include numbers along axes only for bottom left plot of group (a 'group' is an analysis of identical markers).
- [x] All plots are contour plots with outliers or pseudocolor plots.
- [x] A numerical value for number of cells or percentage (with statistics) is provided.

Methodology

Sample preparation	Following anesthesia in continuous flow of 3% isoflurane, blood was collected from the retroorbital plexus via a microcapillary tube into Eppendorf tubes containing 200 µl PBS with 5 mM EDTA. Blood samples were underlayered with 1 ml of lymphocyte separation medium and centrifuged for 25 minutes at 800 g at room temperature, after which the mononuclear cell layer was recovered by aspiration.<br>Spleens and lymph nodes were harvested from freshly euthanized mice, transferred into ice-cold FC buffer (PBS, 2% FCS, 2 mM EDTA), and mechanically dissociated using a pestle, then filtered through a 70 µm cell strainer. The cells were recovered by centrifugation at 200 g for 5 minutes at 4°C. For spleen samples, erythrocytes were lysed by adding RBC lysis buffer (155 mM NH4Cl, 12 mM, NaHCO3, 0.1 mM EDTA) followed by incubation at room temperature for 2-3 minutes. Ice-cold FC buffer was added and the splenocytes were recovered by centrifugation as before and finally resuspended in ice-cold FC buffer before proceeding with staining and analysis.
Instrument	NovoCyte Quanteon 4025 (Agilent) and LSR Fortessa (BD).
Software	Acquisition: Agilent NovoExpress v. 1.5.0 and BD FACSDiva v. 8.0<br>Analysis: FlowJO v. 10.8.0, FCSExpress v. 7
Cell population abundance	No sorting was performed.
Gating strategy	The gating strategies are presented in Supplementary Table 2, as follows:<br><br>All cells are gated as leukocytes (based on FSC-A vs SSC-A plot), single cells (based on FSC-A vs FSC-H and SSC-A vs SSC-H), and live (based on being negative for the Fixable Viability Dye), followed by subset markers:<br><br>Subset / Markers<br>B cell / B220 positive<br>CD4 T cell / CD4 positive<br>CD8 T cell / CD8 positive<br>GC B cell / B220 positive, CD38 low and CD95 positive<br>Plasmablasts (PB) / CD138 positive, B220 high<br>Plasma cells (PC) / CD138 positive, B220 low<br>PB/PC / CD138 positive, B220 positive<br>NK cells / CD4, B220 and CD8 negative. NK1.1 positive<br>Ly6g/c cells / CD4, B220 and CD8 negative. Ly6g/c positive<br>Dendritic cells / CD4, B220 and CD8 negative. NK1.1 and Ly6g/c negative. CD11c positive<br><br>Figures summarizing gating strategies have additionally been provided in the Supplementary Information as Supplementary Figures 9-12.

☒ Tick this box to confirm that a figure exemplifying the gating strategy is provided in the Supplementary Information.
